# Teacher career calling reduces burnout: The mediation effects of work engagement and psychological capital

**DOI:** 10.3389/fpsyg.2022.988467

**Published:** 2022-11-03

**Authors:** Xuan Zhao, Kejia Wu, Binghai Sun, Weijian Li

**Affiliations:** School of Teacher Education, Zhejiang Normal University, Jinhua, China

**Keywords:** career calling, work engagement, burnout, teacher psychological capital, teachers

## Abstract

Burnout is a serious problem in the teaching profession. Research suggests that career calling could be regarded as a protective factor against burnout; however, the mediating mechanism underlying this relationship remains to be explored. The purpose of this study was to test the mediating roles of work engagement and teachers' psychological capital. A total of 3,300 teachers completed a self-report questionnaire. Results showed that the relationship between career calling and burnout was mediated by work engagement and teacher psychological capital. These findings provide insights for preventing burnout among teacher groups.

## Introduction

Burnout, which refers to a series of physiological syndromes that manifest in the occupational environment as a result of stress reactions to chronic sources of emotional and interpersonal tension, has attracted the interest of both researchers and practitioners (Maslach et al., 2001). Teachers who experienced burnout tend to lose patience and compassion for their students, become less prepared for lessons and feel less in control of their work and less accomplished (Shen et al., 2015; Yu et al., 2015). Furthermore, teachers who experienced burnout could also be less enthusiastic about the consequences on teaching quality (Moè, 2016; Frenzel et al., 2019; Lazarides et al., 2021). One survey conducted by Lu (2001) found that primary and secondary school teachers in China are facing greater psychological stress, and this psychological stress has caused a certain negative impact on teaching (e.g., burnout) and students' development (Lu, 2001). Some previous studies have explored several factors that can affect teachers' burnout, such as self-efficacy (Pas et al., 2012; Gillet et al., 2022), emotion regulation (Bing et al., 2022), self-compassionate (Moè and Katz, 2020), teachers' life-responsive beliefs (Pishghadam et al., 2014), time perspective (Meidani et al., 2021) and anxiety (Pressley, 2021). However, little was known about the positive effect of career calling on teachers' burnout and it's mediating mechanisms. Given the negative consequences caused by burnout, it's necessary to explore the potential protective factors against burnout among teachers.

### Career calling and burnout

Career calling could be regarded as a protective factor against burnout. It is defined as a transcendent calling that comes from the self and goes beyond, a way to live out a particular life role in a way that demonstrates or gains a sense of purpose or meaning, as well as other-oriented values and goals as the basic source of motivation (Dik and Duffy, 2009). Research has shown that burnout among teachers can be predicted by the strength of career calling (Hagmaier et al., 2013). For example, one research found that people who regarded their work as their calling were able to adaptively deal with tensions and role conflicts (Oates et al., 2005) and suffer less stress and depression (Treadgold, 1999). According to expectancy-valence theory (Kominis and Emmanuel, 2007) extended by Lewin's (1938) social-behavioral motivation analysis and Tolman's (1951) cognitive-based model of motivation, career calling meant that individuals' work was driven by a deep internal motivation (Duffy et al., 2018; Lysovaa et al., 2019). This intrinsic motivation can reduce their feelings of burnout (Lian et al., 2021). Based on the theory and empirical evidence, this research proposed that burnout among teachers is negatively predicted by career calling.

### Career calling, work engagement, and burnout

The relationship between career calling and burnout may be mediated by work engagement. Work engagement was defined as the control of the ego of an organizational member to integrate the ego with the work role (Kahn, 1990). It refers to the positive, fulfilling, energetic, dedicated, and focused psychological state of an individual toward work (Wu et al., 2014). According to the dual continuum model of motivation proposed by Pishghadam et al. (2021), individual active motivation and high career calling mean high work engagement, while the lack of work engagement turns into active demotivation, manifested as burnout. An important psychological characteristic of a career calling is an action orientation, a willingness to go the extra mile for the job (Xie et al., 2016). And it is a very strong intrinsic motivation that leads individuals to engage in work they like, actively search for relevant information, and improve their abilities (Dobrow and Tosti-Kharas, 2012). Therefore, there should be a close relationship between career calling and work engagement. One research conducted by Seco and Lopes (2013) found work engagement was positively predicted by career calling (Seco and Lopes, 2013).

Meanwhile, previous studies have examined the link between work engagement and burnout. Although they are both parts of occupational mental health, they are the opposite of each other (Qi et al., 2016). Maslach and Leiter (1997) argue that job burnout is the erosion of commitment to work that makes otherwise important, meaningful, and challenging work unpleasant, meaningless, and unfulfilling (Wang and Qin, 2009). However, actively engaging in an activity requires a large amount of energy, attention, and concentration, which can counteract burnout to some extent (Pishghadam et al., 2019a). Some previous studies have found that there is a negative relationship between burnout and work engagement (Schaufeli et al., 2002). Thus, we proposed that work engagement may mediate the direct link between career calling and burnout.

### Career calling, teacher psychological capital, and burnout

The relationship between career calling and burnout may also be mediated by the teacher's psychological capability. In the context of the positive psychology movement, Luthans et al. (2005) introduced the concept of psychological capital (Luthans et al., 2005), which can be defined as the core psychological element of general individual motivation, enabling individuals to gain a competitive advantage through targeted input and development.

Psychological capital is an upward and positive psychological state possessed by an individual, which is a potentially available intra-individual resource that can help individuals cope with difficulties and setbacks, and promote their growth and development (Luthans et al., 2007; Cheng et al., 2020). Based on the work as calling theory (Duffy et al., 2018), calling can represent meaningful experience and positive attitudes toward one's work. Calling comprises positive psychological capability, especially those that manifest positive emotion and attitude (Lian et al., 2021). Numerous studies have shown that career calling shows significant positive relationships with psychological capability such as life meaning experience, life satisfaction, and academic satisfaction (Zhang et al., 2013). From previous studies on psychological capital and burnout among elementary school teachers, civil servants, and college students, burnout was found to be negatively related to psychological capital (Liu and Fu, 2013). According to Hobfoll's resource conservation theory (Hobfoll, 2001), people always try to acquire and maintain the resources they consider valuable (including conditions, time, ability, etc.), and psychological capital, as a personal resource, helps individuals cope with work stress and reduce burnout (Hobfoll, 2001). Thus, this study proposed that the relationship between career calling and burnout is mediated by teacher psychological capability.

### Career calling, work engagement, teacher psychological capital, and burnout

Furthermore, this study proposed that this relationship can be mediated by work engagement and teacher psychological capital sequentially. Specifically speaking, career calling leads to high levels of work engagement, enhancing teacher psychological capital, thus reducing the level of burnout. The research operated by Seco and Lopes (2013) found that career calling was positively related to work engagement. Mao and Xie (2013) found that the positive correlation between teachers' psychological capital and work engagement was significant, and psychological capital positively predicted the variation of work engagement. Furthermore, previous research has found that teacher psychological capability negatively predicts burnout (Hobfoll, 2001). Thus, the relationship between career calling and burnout may be sequentially mediated by work engagement and teacher psychological capital.

### Aims of this study

**Figure 1:**
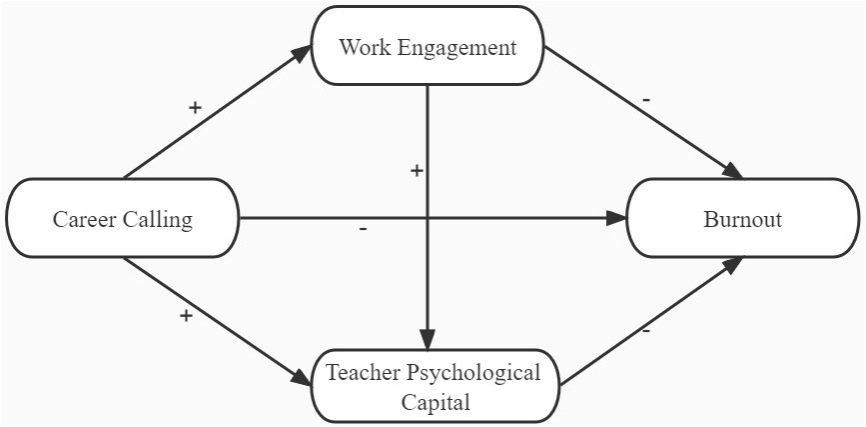
The hypothesized model.

The principal motivation behind this study is twofold. First, this study aims to investigate the connection between career calling and burnout. Second, considering the analysis and confirmations of past research, we intend to test whether work engagement and teachers' psychological capital play parallel and multiple mediators. Based on the theory and the existing empirical results, we hypothesize that: (H1) Burnout is negatively predicted by career calling, (H2 and H3) work engagement and teacher's psychological capital play a mediating role, and (H4) work engagement and teacher's psychological capital play a chain-mediating role. Figure 1 displays all the assumptions.

## Methods

### Participants

This study was approved by the local ethics committee and conducted by the Declaration of Helsinki and APA ethical standards. Data was collected online. And informed consent was also provided to participants. All analyses were conducted with SPSS 21.0.

In the beginning, the data from 3,300 teachers from a city in Zhejiang Province were obtained. After screening, 2,929 valid responses (response rate: 89%) were included for further analysis. The final sample includes 853 male (29.1%) and 2,076 female respondents (70.9%), with an average age of 39 years (*SD* = 8.67). Teachers of kindergarten totaled 336 (11.5%); of primary school, 1,601 (54.7%); middle school, 675 (23.0%); and high school, 317 (10.8%). Participants had been teaching for an average of 17.90 years (*SD* = 9.97).

### Measures

#### Career calling

The Subjects' career calling was measured by the Chinese version of the career calling scale, which was adapted from the scale developed by Dobrow and Tosti-Kharas (2012). The scale consists of 12 items (e.g., “I am passionate about what I do”). All items were answered on a seven-point Likert scale (1 = not at all, 7 = very much). Higher scores implied a higher level of career calling. In this study, the Cronbach's α for the scale was 0.96. χ^2^/*df* = 3.412, CFI = 1.000, AGFI = 0.985, TLI = 0.996, RMSEA = 0.029 (90% CI = 0.020, 0.038), indicating the tool has a good validation.

#### Work engagement

Work engagement was assessed by the UWES9 Scale, which was developed by Bakker et al. (2000). The questionnaire included 9 items, such as “I feel myself bursting with energy in my work”. Participants were required to answer on a five-point scale, which ranged from 1 (strongly disagree) to 5 (strongly agree). Higher scores indicated a higher level of engagement in putting in the work. In this study, Cronbach's α for the scale was 0.94. χ^2^/*df* = 2.410, CFI = 1.000, AGFI = 0.992, TLI = 0.998, RMSEA = 0.022 (90% CI = 0.000, 0.048), which indicates a good validity.

#### Teacher psychological capital

Teacher Psychological Capital was using the Psychological Capital Questionnaire for Primary and Secondary School Teachers developed by Zhang (2010). The scale includes 19 items, such as “When I encounter difficulties in teaching, I am often at a loss as to what to do”, and is scored on a 6-point scale from 1 (completely disagree) to 6 (completely agree), with higher scores indicating higher levels of organizational support for the individual. The Cronbach alpha coefficient of the scale in this study was 0.93. χ^2^/*df* = 3.281, CFI = 0.995, AGFI = 0.978, TLI = 0.988, RMSEA = 0.028 (90% CI = 0.024, 0.032), indicating the validity of the tool was good.

#### Burnout

Burnout was measured by the Professional Quality of Life Scale designed by Stamm (2010), which includes three dimensions: compassion satisfaction, burnout, and secondary traumatic stress. The subscale of burnout includes eight items rated on a five-point Likert scale (1 = never, 5 = very often), with higher scores indicating higher levels of burnout. The Cronbach alpha for the scale was 0.90. χ^2^/*df* = 4.365, CFI = 0.999, AGFI = 0.985, TLI = 0.989, RMSEA = 0.034 (90% CI = 0.013, 0.058), indicating the tool we used in this study has good validity.

### Analytic strategy

Data analyses were performed using SPSS and the SEM module based on JASP (Goss-Sampson, 2019). First, descriptive statistics and correlation analyses were conducted on the main variables. Second, the structural equation model module was used to test the model. The parameters of the structural equation model analysis were set as follows: standardized estimates, bootstrap with replications (set at 1,000), emulation with Mplus, and estimator was set as maximum likelihood.

## Results

### Preliminary analyses

Means, standard deviations, and Pearson's correlations among variables were calculated and are shown in Table 1. Career Calling was positively associated with Work Engagement, as well as with Teacher Psychological Capital. Work Engagement was positively correlated with Teacher Psychological Capital. Burnout was negatively associated with Career Calling, Work Engagement, and Teacher Psychological Capital. Years of teaching were positively correlated to Career Calling, Work Engagement, and Psychological Capital.

**Table 1:** Means, standard deviations, and Pearson's correlations among variables.

**Variables**	***M***	***SD***	**YT**	**CC**	**WE**	**TPC**	**BO**
YT	17.90	9.97	–				
CC	5.66	1.13	0.06**	–			
WE	3.90	0.78	0.09**	0.75**	–		
TPC	4.78	0.75	0.04*	0.71**	0.73**	–	
BO	2.16	0.70	0.02	−0.65**	−0.67**	−0.77**	–

YT, years of teaching; CC, career calling; WE, work engagement; TPC, teacher psychological capital; BO, burnout.

** p < 0.01.

We compared the score of teacher burnout among kindergarten, primary school, secondary school, and high schools with One Way ANOVA analysis. The results of ANOVA were significant (*F*_(3,2925)_ = 10.04, *p* < 0.000). The results of *Post hoc* analysis showed that the score of burnout among kindergarten teachers (*M* = 2.00, *SD* = 0.71) was smaller than primary school teachers (*M* = 2.17, *SD* = 0.70, *p* < 0.000), secondary school teachers (*M* = 2.24, *SD* = 0.71, *p* < 0.000), and high school teachers (*M* = 2.24, *SD* = 0.71, *p* < 0.000), and the score of burnout among primary, secondary and high school teachers has no difference.

### The multiple mediation models

**Figure 2:**
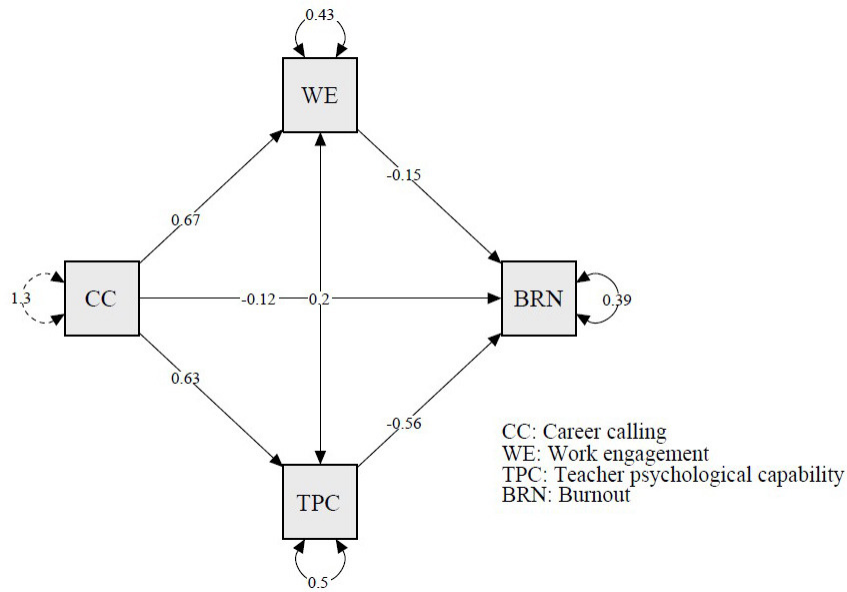
The results of sequential mediation analysis.

A structural equation model based on the SEM module of JASP was used to test the multiple mediation model (Goss-Sampson, 2019). Results showed that the pathways for the direct effect were significant (career calling → burnout), which supports the H1. Moreover, the indirect effects *via* the work engagement and teacher psychological capital were also significant (i.e., “career calling → work engagement → burnout” and “career calling → teacher psychological capital → burnout”). Hence, the H2 and H3 were supported. In addition, we also detected a significant result in the pathway (career calling → work engagement → teacher psychological capital → burnout), which supported the H4. Thus, the multiple mediation model was proved to be effective. The results of the mediation analyses are shown in Figure 2.

## Discussion

### Different levels of burnout among different teachers' group

This study found that compared with kindergarten teachers, the level of burnout was higher among primary, secondary, and high school teachers. This result was similar to other previous studies (Wu and Zheng, 2012; Li et al., 2015). One possible explanation for this difference may be that compared to kindergarten teachers, primary, secondary, and high school teachers would face more heavy teaching tasks due to that they should get more of their students into good middle schools, high schools, and colleges. Thus, they will experience more pressure and their level of burnout would be higher.

### Relationship between career calling and burnout

This study found that burnout was negatively predicted by career calling, supporting H1. The results were similar to previous findings. For instance, Zhang et al. (2020) found that among Chinese physicians, suffering from high levels of burnout threatened their career calling. Additionally, our results validated the Work as Calling Theory (Duffy et al., 2018), suggesting that career calling can represent meaningful experience and positive attitudes toward one's work, which can arouse more positive emotions and then reduce feelings of burnout. According to the research on stroke in language learning by Pishghadam and Karami (2017), teachers with high career calling will increase their degree to stroke students, which will affect students' evaluation of their level of success, to alleviate their burnout.

### The mediating effect of work engagement

We found that work engagement played a mediating role in this relationship. Specifically speaking, teachers with high career calling are more likely to engage themselves in work, which indirectly reduces burnout. This result was consistent with previous findings (Schaufeli et al., 2002; Seco and Lopes, 2013) that career calling is positively related to work engagement, and burnout is negatively correlated with work engagement. This result can be explained as follows. Career calling provides individuals with a sense of meaning and identity at work, making people more engaged in their work (Duffy et al., 2012). In addition, Dobrow and Tosti-Kharas (2011) confirmed a significant correlation between career calling and work engagement in an empirical survey of managers (Dobrow and Tosti-Kharas, 2011). Additionally, some researchers consider work engagement can be seen as the antipode of burnout (Sweetman and Luthans, 2010). According to Pishghadam (2016) research on emotioncy, teachers' high career calling will improve their received emotions and sensory input, which will affect teachers' work engagement, their understanding of reality, their perception of the future, and their perception of their career, that is, it will affect the level of burnout (Miri and Pishghadam, 2021; Pishghadam et al., 2022).

Previous studies have developed the theory of the structure of employee well-being, confirming the dichotomy between work engagement and burnout in terms of energy and identity dimensions, that is, lower work engagement means lower energy and dedication of the individual, which leads to emotional exhaustion and cynicism, ultimately leading to higher burnout (Maslach and Leiter, 1997; Schaufeli et al., 2002). Moreover, Relativism showed that sensory experience will affect our emotions. Based on different exploration and participation in the profession, teachers will have different degrees of career calling and develop different degrees of burnout (Pishghadam et al., 2016).

Hence, individuals with low career calling are more likely to work without engagement. Teachers who are prone to working with engagement tend to be energetic and enthusiastically involved in their work (Bakker et al., 2008), reducing burnout. Hence, the relationship between career calling and burnout may be mediated by work engagement.

### The mediating effect of teacher psychological capital

This study found that teachers' psychological capital also played a mediating role in the relationship. Some previous findings can support this result (Hobfoll, 2001). According to the concept of Pishghadam et al. (2019b) sensory capital, teachers' psychological capital can be promoted by sensory capital, which can enhance teachers' emotional consciousness and improve their career calling. According to the Job Demands-Resources (JD-R) model of burnout (Bakker and Demerouti, 2007), strong job resources can reduce the level of job burnout to a certain extent, and calling may be an internal psychological resource that has motivational functions (Dicke et al., 2018; Nahrgang et al., 2011). Therefore, psychological capital, as an important personal resource, can be regarded as a special work resource to alleviate burnout. Additionally, the existing onion model of a good teacher (Korthagen, 2004) asserts that the deepest core feature of a good teacher is career calling. Unsurprisingly, high career calling can improve work motivation and improve teachers' psychological capital. As a result, they become more willing to work hard and are less likely to experience burnout. This is the reason why teacher psychological capital could mediate the relationship between career calling and burnout.

### The sequential mediating effects of work engagement and teacher psychological capital

A notable finding of this study was that the relationship between career calling and burnout was meditated by work engagement and teacher psychological capital sequentially, supporting H4. This finding was consistent with previous findings (Duffy et al., 2018). This implies that Chinese teachers with a high level of career calling usually tend to invest themselves in work, which enhances their psychological capital as teachers, ultimately decreasing their burnout.

Although little research directly tested the mediating roles of work engagement and teacher psychological capital between career calling and burnout, some previous results indirectly support the finding of this study. The sequential mediation model shows that work engagement is positively correlated with teacher psychological capital, which is consistent with the findings of previous studies (Mao and Xie, 2013). An explanation was that work engagement enhances teachers' life meaning experience, life satisfaction, and academic satisfaction (Zhang et al., 2013), which leads to elevated psychological capital. Work engagement commonly refers to a positive, fulfilling emotional and cognitive state related to work (Li and Ling, 2007). And the career calling happens to provide a sense of meaning and identity at work. Thus, individuals with high levels of occupational calling are more likely to be engaged in their work and to have more positive psychological capital, which reduces burnout.

### Educational implications

This study reveals the mediating mechanisms underlying the relationship between career calling and burnout. In addition, this study has significant practical implications. Firstly, this study found that teacher career calling has a positive effect on preventing burnout. Thus, the utilization and cultivation of career calling could be used to design interventions to prevent burnout. On the one hand, school administrators could make teachers focus on their career de elopement; on the other hand, administrators could help teachers tap into their inner voices and make them feel the meaning and value of their work through all kinds of activities. Secondly, based on the findings of this study, boosting teachers' work engagement and teacher psychological capability should also be considered an effective way to reduce burnout. For example, school administrators should talk with teachers about their work engagement and teacher psychological capability, to avoid burnout. Specifically speaking, the education sector should provide primary and secondary school teachers with more abundant learning resources, pay more attention to the teachers' psychological development, and appropriately increase the content of teachers' psychological construction in teacher professional training, all of these manners may be effective to increase work engagement and supply psychological capital for teachers.

## Limitations and future directions

This study has several limitations. First, the cross-sectional survey design we used had difficulties in concluding the causality. The longitudinal or experimental design may be used in future research. Second, the sample in our study was from the same city, resulting in deficits in generalizing the results. Future research should choose a more representative sample to investigate teachers' burnout.

## Conclusion

This study found that the relationship between career calling and burnout was mediated by work engagement and teacher psychological capital in a parallel and sequential manner. This study suggests that strengthening work engagement and teacher psychological capital may effectively help teachers reduce burnout.

## Data availability statement

The raw data supporting the conclusions of this article will be made available by the authors, without undue reservation.

## Ethics statement

The studies involving human participants were reviewed and approved by Zhejiang Normal University. The patients/participants provided their written informed consent to participate in this study.

## Author contributions

WL, BS, and XZ: conceptualization. KW: methodology. WL and BS: resources and project administration. XZ and KW: writing—original draft preparation and writing—review and editing. WL: supervision and funding acquisition. All authors contributed to the article and approved the submitted version.

## Funding

This research was funded by the National Natural Science Foundation of China (grant number 31871124).

## Conflict of interest

The authors declare that the research was conducted in the absence of any commercial or financial relationships that could be construed as a potential conflict of interest.

## Publisher's note

All claims expressed in this article are solely those of the authors and do not necessarily represent those of their affiliated organizations, or those of the publisher, the editors and the reviewers. Any product that may be evaluated in this article, or claim that may be made by its manufacturer, is not guaranteed or endorsed by the publisher.
